# A glycolysis-related two-gene risk model that can effectively predict the prognosis of patients with rectal cancer

**DOI:** 10.1186/s40246-022-00377-0

**Published:** 2022-02-02

**Authors:** Zhenzhen Liu, Zhentao Liu, Xin Zhou, Yongqu Lu, Yanhong Yao, Wendong Wang, Siyi Lu, Bingyan Wang, Fei Li, Wei Fu

**Affiliations:** 1 Department of General Surgery, Peking University Third Hospital, 49 Huayuan North Road, Haidian District, Beijing, People’s Republic of China; 2 Department of Medical Oncology and Radiation Sickness, Peking University Third Hospital, Beijing, People’s Republic of China

**Keywords:** Glycolysis, Rectal cancer, *TSTA3*, *PKP2*, Prognosis

## Abstract

**Background:**

Aerobic glycolysis is an emerging hallmark of cancer. Although some studies have constructed glycolysis-related prognostic models of colon adenocarcinoma (COAD) based on The Cancer Genome Atlas (TCGA) database, whether the COAD glycolysis-related prognostic model is appropriate for distinguishing the prognosis of rectal adenocarcinoma (READ) patients remains unknown. Exploring critical and specific glycolytic genes related to READ prognosis may help us discover new potential therapeutic targets for READ patients.

**Results:**

Three gene sets, HALLMARK_GLYCOLYSIS, REACTOME_GLYCOLYSIS and REACTOME_REGULATION_OF_GLYCOLYSIS_BY_FRUCTOSE_2_6_BISPHOSPHATE_METABOLISM, were both significantly enriched in both COAD and READ through glycolysis-related gene set enrichment analysis (GSEA). We found that six genes (*ANKZF1, STC2, SUCLG2P2, P4HA1, GPC1* and *PCK1*) were independent prognostic genes in COAD, while *TSTA3* and *PKP2* were independent prognostic genes in READ. Glycolysis-related prognostic model of COAD and READ was, respectively, constructed and assessed in COAD and READ. We found that the glycolysis-related prognostic model of COAD was not appropriate for READ, while glycolysis-related prognostic model of READ was more appropriate for READ than for COAD. PCA and t-SNE analysis confirmed that READ patients in two groups (high and low risk score groups) were distributed in discrete directions based on the glycolysis-related prognostic model of READ. We found that this model was an independent prognostic indicator through multivariate Cox analysis, and it still showed robust effectiveness in different age, gender, M stage, and TNM stage. A nomogram combining the risk model of READ with clinicopathological characteristics was established to provide oncologists with a practical tool to evaluate the rectal cancer outcomes. GO enrichment and KEGG analyses confirmed that differentially expressed genes (DEGs) were enriched in several glycolysis-related molecular functions or pathways based on glycolysis-related prognostic model of READ.

**Conclusions:**

We found that a glycolysis-related prognostic model of COAD was not appropriate for READ, and we established a novel glycolysis-related two-gene risk model to effectively predict the prognosis of rectal cancer patients.

**Supplementary Information:**

The online version contains supplementary material available at 10.1186/s40246-022-00377-0.

## Introduction

Colorectal cancer (CRC), including colon cancer and rectal cancer, is the third most commonly diagnosed malignancy and ranks second in terms of mortality among cancers globally [1, 2]. It is estimated that the incidence of CRC will increase to 2.5 million new cases worldwide in 2035 due to the constant increase in developing countries and increased morbidity in younger people [3]. Although colon cancer and rectal cancer have been regarded as the same disease over the past decades, accumulating evidence has revealed that rectal cancer is different from colon cancer in many aspects, such as embryological origins, anatomy, risk factors, sensitivities to carcinogens, microbiota, and genetic subtypes [3–5]. Moreover, rectal cancer is the most common subtype among Asians, and the past decade has witnessed an increasing trend of early-onset rectal cancer (diagnosis before 50 years of age) [2, 4, 6, 7], which has constituted a formidable challenge in China and needs further investigation [4, 8]. Various biomarkers associated with the survival and prognosis of rectal cancer have been explored in the past. However, a single gene or biomarker cannot accurately predict the outcomes of cancer patients. The past decade has witnessed the soaring development of high-throughput sequencing [9–12], which has expanded our insight into the genetic alterations of rectal cancer and made it possible to establish multiple-gene predictive models using clinical and genetic data obtained from the public databases [13–15]. An mRNA-based gene signature for predicting rectal cancer patient outcomes is still needed in the present context.

Reprogramming energy metabolism is an emerging hallmark of cancer [16]. Even in the presence of oxygen, tumor cells give priority to glycolysis rather than mitochondrial oxidative phosphorylation for glucose catabolism, resulting in a state termed “aerobic glycolysis” or the “Warburg effect” [17]. A well-acknowledged rationale for the glycolytic switch in tumor cells is that increased glycolysis is employed by proliferating cells as a versatile product line, which continuously generates glycolytic intermediates for various biosynthetic pathways to satisfy the requirement for active cell proliferation [18]. Thus, exploring critical glycolytic genes related to rectal cancer prognosis may help us discover new potential therapeutic targets.

In this study, we found that a glycolysis-related prognostic model of colon cancer could not distinguish the prognosis of rectal cancer patients through bioinformatic analysis based on The Cancer Genome Atlas (TCGA) database. Moreover, we discovered the critical and specific glycolysis-related genes that participate in the development of rectal cancer using bioinformatic methods and established a practical model to predict rectal cancer patients' prognosis.

## Materials and methods

### Study design

Our study compared glycolysis-related gene sets through gene set enrichment analysis (GSEA) and compared glycolysis-related independent prognosis genes between colon adenocarcinoma (COAD) and rectal adenocarcinoma (READ). Glycolysis-related prognostic model of COAD and READ was, respectively, constructed and assessed in COAD and READ. Assessment of the glycolysis-related READ prognostic model in READ patients was further performed through time-dependent receiver operating characteristic curve (time ROC) analysis, univariate and multivariate Cox analyses, principal component analysis (PCA) analysis, t-distributed stochastic neighbor embedding (t-SNE) analysis, Gene Ontology (GO) enrichment and Kyoto Encyclopedia of Genes and Genomes (KEGG) analyses. A nomogram combining the risk model of READ with clinicopathological characteristics was established for READ. The workflow for this study is shown in Additional file 1: Fig. S1.

### Data collection

The mRNA expression profile of 144 READ and 8 adjacent normal rectal tissues were downloaded from the TCGA Genomic Data Commons (GDC) database (https://portal.gdc.cancer.gov/). The mRNA expression profiles of 398 COAD and 39 adjacent normal colon tissues were also downloaded from the TCGA GDC database. The mRNA profiles were standardized by log2 transformation for further analysis. However, only 142 rectal cancer samples were documented with mRNA expression profiles and detailed clinicopathological information, including age, gender, American Joint Committee on Cancer (AJCC) TNM stage, T stage, N stage, M stage and survival status. The detailed clinical features of 142 rectal cancer patients are shown in Table 1. Patients whose follow-up time was absent, zero-days or unknown were excluded from the survival analysis. Finally, 135 READ patients and 363 COAD patients were enrolled for further survival analysis of glycolysis-related genes.

**Table 1:** Clinical features in READ patients

Variables	Patients, *n* (%)
Sex	
Male	77 (54.2%)
Female	65 (45.8%)
Age, years	
≤ 65	73 (51.4%)
> 65	69 (48.6%)
TNM stage	
I	26 (18.3%)
II	41 (28.9%)
III	45 (31.7%)
IV	22 (15.5%)
Unknown	8 (5.6%)
T stage	
T1	7 (4.9%)
T2	25 (17.6%)
T3	98 (69.0%)
T4	11 (7.8%)
Unknown	1 (0.7%)
N stage	
N0	70 (49.2%)
N1	40 (28.2%)
N2	29 (20.4%)
N3	0 (0.0%)
NX	2 (1.4%)
Unknown	1 (0.7%)
M stage	
M0	106 (74.6%)
M1	21 (14.9%)
MX	13 (9.1%)
Unknown	2 (1.4%)
Follow up time	
= 0 day or unknown	7 (4.9%)
> 0 day	135 (95.1%)

### Gene set enrichment analysis (GSEA)

Gene sets enrichment analysis (GSEA) was conducted by GSEA software 4.10 from the Broad Institute [19] to identify the gene sets enriched between cancer tissues and adjacent normal tissues. A total of 12 glycolysis-related gene sets were downloaded from the Molecular Signatures Database (MSigDB) (https://www.gsea-msigdb.org/gsea/msigdb/genesets.jsp). To acquire a normalized enrichment score (NES), we conducted 1000 times gene set permutations for each analysis. When |NES| > 1, nominal *p* value < 0.1 and false discovery rate (FDR) *q* value < 0.1, the investigated gene sets were considered statistically significant. Glycolysis-related genes were extracted from the analysis performed by GSEA. We utilized the “limma” R package for analysis, which in turn yielded glycolysis-related differentially expressed genes (DEGs), with filtering criteria of *p* value < 0.05 and log2 fold change (log2FC) ≠ 0.

### Standardization of data processing and construction of the risk score model

Univariate Cox regression analysis was first performed to screen out genes that were associated with overall survival (OS) (*p* value < 0.05). Subsequently, the identified genes were subjected to multivariate Cox regression analysis, and the corresponding regression coefficients related to OS were also acquired. When the values of hazard ratio (HR) are larger than 1, the corresponding factor is considered a risk factor. We used the “Cox. Zph” function to test the Cox proportional risk model in the “survival” R package. When the p value of each covariate and global test is > 0.05, it is considered that the Cox model conforms to the proportional risk hypothesis. In this study, glycolysis-related gene risk conformed to the proportional risk hypothesis and was constructed using the “coxph” function in the “survival” R package. The risk score formula is as follows: ${\text{Risk}}\;{\text{score}} = \mathop \sum \nolimits_{i = 1}^{n} {\text{expression}}\;{\text{of}}\;Gi \times \beta i$ (“*G*” means gene; “*i*” means order of genes; “*n*” means the number of prognostic genes; “β” means the regression coefficient of the corresponding gene after multivariate Cox analysis). According to the median risk score, all the included patients were divided into either the high-risk group and low-risk group.

### Assessment of the risk score model in rectal cancer patients

Kaplan–Meier survival curves were drawn to show the survival rate difference between the two groups with a *p* value < 0.05 considered statistically significant. A ROC curve was plotted to evaluate the sensitivity and specificity of the risk score model. When the area under the curve (AUC) is 0.5–0.7, the risk score model has acceptable efficiency. When the AUC is larger than 0.7, the risk score model has good accuracy. The “ggplot2” and “pheatmap” R packages were used to plot heat maps and survival status charts. PCA and t-SNE were performed to explore the distribution of different groups using the “Rtsne” R package. The “rms” R package was used to plot the nomogram for predicting the survival of rectal cancer patients. The time-dependent ROC curves and calibration plots were depicted to evaluate the efficiency of the nomogram.

### Functional enrichment analysis

The “clusterProfiler” R package was utilized to conduct Gene Ontology (GO) and Kyoto Encyclopedia of Genes and Genomes (KEGG) analyses based on the DEGs (|Log2FC| > 0.6, FDR < 0.05) between the high-risk and low-risk groups. *P* values were adjusted with the Benjamini–Hochberg (BH) method.

### Statistical analysis

All relevant statistical analyses were performed with R software (*R4.0.2*, https://www.r-project.org/).

## Result

### GSEA of glycolysis-related genes in COAD and READ

GSEA was, respectively, conducted to explore whether glycolysis-related gene sets were significantly enriched in the colon cancer and rectal cancer. Twelve glycolysis-related gene sets were investigated. Five gene sets, highlighted in boldface in Table 2, HALLMARK_GLYCOLYSIS, REACTOME_GLYCOLYSIS, REACTOME_REGULATION_OF_GLYCOLYSIS_BY_FRUCTOSE_2_6_BISPHOSPHATE_METABOLISM, BIOCARTA_FEEDER_PATHWAY and BIOCARTA_GLYCOLYSIS_PATHWAY, were significantly enriched in the colon cancer samples (|NES| > 1, nominal *p* value < 0.1 and FDR *q* value < 0.1), as shown in Table 2. Four gene sets, highlighted in boldface in Table 2, HALLMARK_GLYCOLYSIS, REACTOME_GLYCOLYSIS, GO_FRUCTOSE_1_6_BISPHOSPHATE_METABOLIC_PROCESS, and REACTOME_REGULATION_OF_GLYCOLYSIS_BY_FRUCTOSE_2_6_BISPHOSPHATE_METABOLISM, were significantly enriched in the rectal cancer samples (|NES| > 1, nominal *p* value < 0.1 and FDR *q* value < 0.1), as shown in Table 2. Three gene sets, HALLMARK_GLYCOLYSIS, REACTOME_GLYCOLYSIS, and REACTOME_REGULATION_OF_GLYCOLYSIS_BY_FRUCTOSE_2_6_BISPHOSPHATE_METABOLISM, were both significantly enriched in the colon cancer and rectal cancer.

**Table 2:** Glycolysis-related GSEA analysis on mRNA expression among COAD and READ patients

The molecular signatures database (MSigDB)	Gene set size	Enrichment score (ES)		Normalized enrichment score (NES)		Nominal *p* value		FDR *q* value		FWER *p* value	
		COAD READ		COAD READ		COAD READ		COAD READ		COAD READ	
**GO_FRUCTOSE_1_6_BISPHOSPHATE_METABOLIC_PROCESS**	10	0.56	0.732	1.22	**1.507**	0.239	**0.036**	0.239	**0.036**	0.128	**0.023**
GO_LACTATE_TRANSMEMBRANE_TRANSPORTER_ACTIVITY	6	− 0.47	− 0.352	− 1.11	− 0.771	0.319	0.804	0.319	0.804	0.126	0.268
GO_LACTATE_TRANSPORT	7	− 0.45	− 0.338	− 1.08	− 0.763	0.336	0.811	0.336	0.811	0.142	0.246
**REACTOME_GLYCOLYSIS**	71	0.67	0.593	**1.98**	**1.568**	**0.002**	**0.033**	**0.002**	**0.033**	**0.001**	**0.019**
**REACTOME_REGULATION_OF_GLYCOLYSIS_BY_FRUCTOSE_2_6_BISPHOSPHATE_METABOLISM**	12	− 0.60	− 0.537	**− 1.53**	**− 1.388**	**0.051**	**0.070**	**0.051**	**0.070**	**0.023**	**0.026**
**HALLMARK_GLYCOLYSIS**	199	0.52	0.436	**1.73**	**1.355**	**0.004**	**0.068**	**0.004**	**0.068**	**0.002**	**0.048**
MODULE_306	26	− 0.38	− 0.319	− 0.82	0.714	0.640	0.784	0.640	0.784	0.338	0.338
KEGG_GLYCOLYSIS_GLUCONEOGENESIS	62	− 0.39	− 0.285	− 1.19	− 0.904	0.264	0.636	0.264	0.636	0.128	0.226
KEGG_CITRATE_CYCLE_TCA_CYCLE	31	− 0.64	− 0.542	− 1.46	− 1.282	0.121	0.203	0.121	0.203	0.059	0.092
**BIOCARTA_FEEDER_PATHWAY**	9	− 0.71	− 0.491	**− 1.52**	− 1.175	**0.089**	0.233	**0.089**	0.233	**0.040**	0.081
BIOCARTA_GLYCOLYSIS_PATHWAY	3	0.89	0.840	1.34	1.261	0.087	0.159	0.087	0.159	0.044	0.082
BIOCARTA_KREB_PATHWAY	8	− 0.73	− 0.694	− 1.35	− 1.344	0.189	0.167	0.189	0.167	0.099	0.081

The significant glycolysis-related gene sets were highlighted in boldface when |NES|>1, and nominal* p* value < 0.1 and false discovery rate (FDR)* q*-value < 0.1

### Comparison of glycolysis-related prognostic genes between COAD and READ

**Fig. 1:**
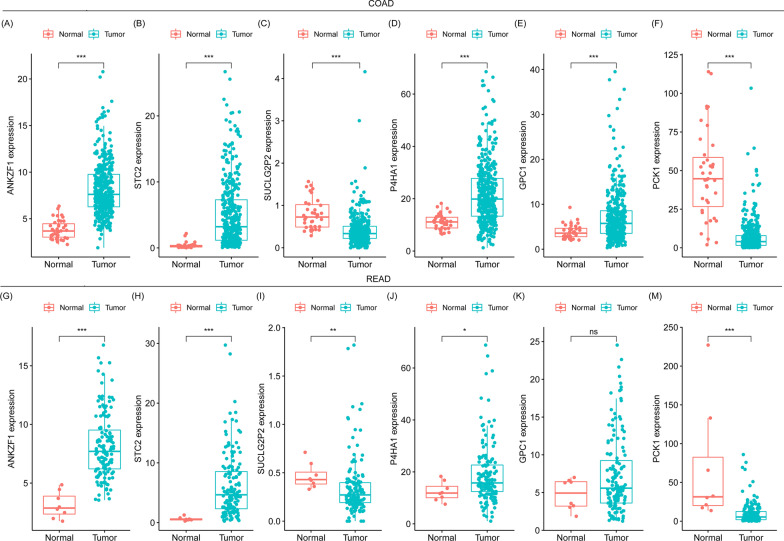
The expression levels of COAD prognostic genes in COAD and READ. **A**–**F** The expression levels of ANKZF1 (**A**), STC2 (**B**), SUCLG2P2 (**C**), P4HA1 (**D**), GPC1 (**E**) and PCK1 (**F**) between cancer and adjacent normal tissue in COAD. (G-M)The expression levels of ANKZF1 (**G**), STC2 (**H**), SUCLG2P2 (**I**), P4HA1 (**J**), GPC1 (**K**) and PCK1 (**M**) between cancer and adjacent normal tissue in READ

**Fig. 2:**
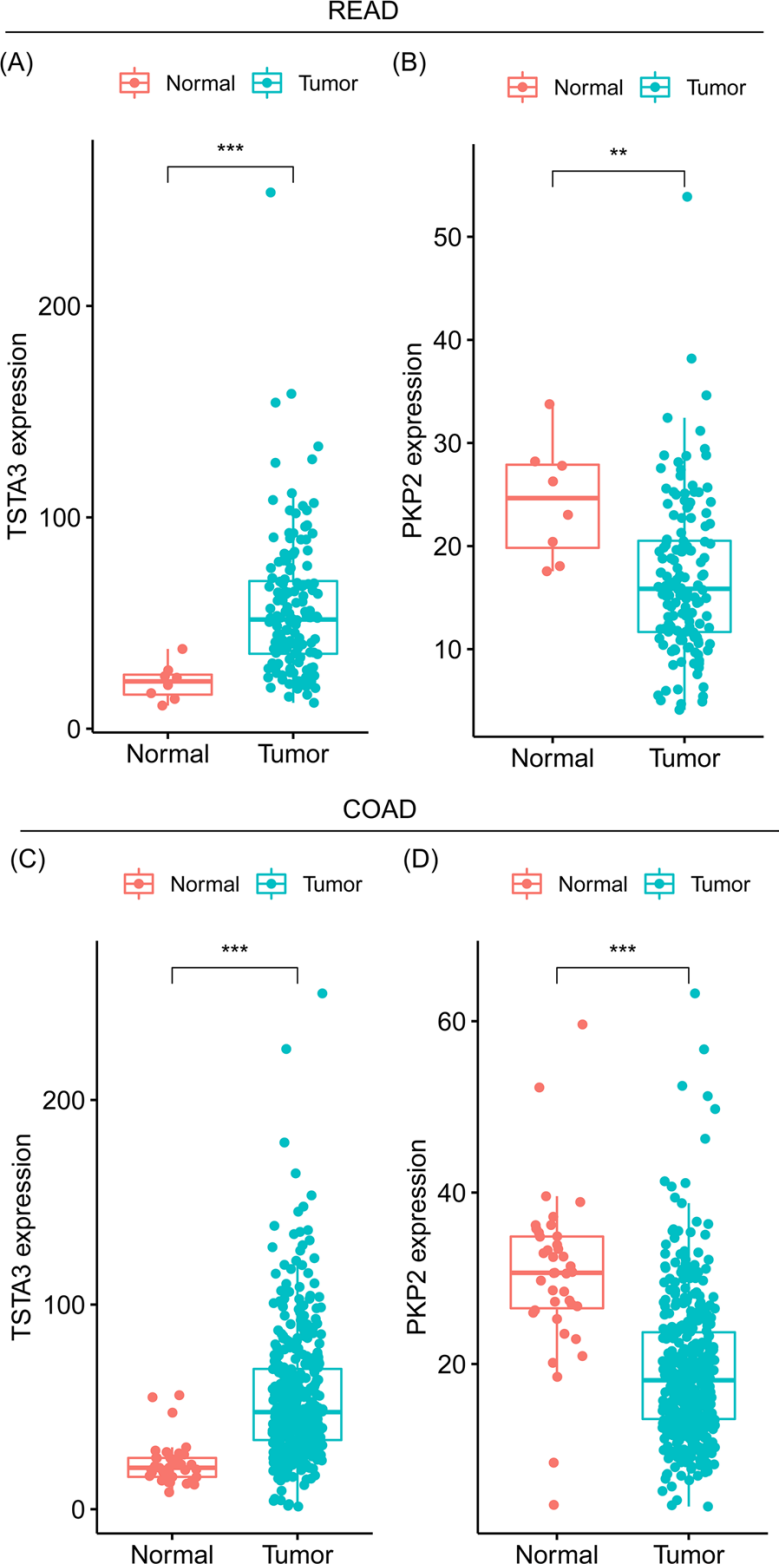
The expression levels of READ prognostic genes in COAD and READ. **A**, **B** The expression levels of TSTA3 (**A**) and PKP2 (**B**) between cancer and adjacent normal tissue in READ. **C**, **D** The expression levels of TSTA3 (**C**) and PKP2 (**D**) between cancer and adjacent normal tissue in COAD

A total of 316 genes in the above gene sets were included for further analysis. Subsequently, 199 glycolysis-related DEGs in READ and 254 glycolysis-related DEGs in COAD were, respectively, identified with *p* value < 0.05 and |log2FC| > 0 as the cutoff standard. To further, respectively, explore prognostic genes in COAD and READ, glycolysis-related DEGS were analyzed by univariate Cox regression. Nine genes (*STC1, ANKZF1, STC2, SDHB, SUCLG2P2, P4HA1, PPFIA4, GPC1* and *PCK1*) in COAD and 4 genes (*TSTA3, IDH3A, PKP2* and *ACO2*) in READ were found to be significantly associated with overall survival (OS) (*p* value < 0.05) (Table 3). No same genes were both significantly associated with OS in COAD and READ. Subsequently, multivariate Cox regression analysis was, respectively, performed in COAD and READ. Six genes including A*NKZF1, STC2, SUCLG2P2, P4HA1, GPC1* and *PCK1* were proven to be independent prognostic factors in COAD (Table 4). Two genes, *TSTA3* and *PKP2*, were proven to be independent prognostic factors in READ (Table 4). We compared the expression levels of the above genes between the cancer tissues and adjacent normal tissues, and found that *ANKZF1, STC2* and *P4HA1* were significantly upregulated, while *SUCLG2P2* and *PCK1* were downregulated in both COAD and READ among the independent prognostic genes of COAD (Fig. 1). Although GPC1 was significantly upregulated in COAD, it was not upregulated in READ (Fig. 1). Among the independent prognostic genes of READ, we found that *TSTA3* was significantly upregulated while *PKP2* was downregulated in both COAD and READ (Fig. 2). Taken together, these results indicated that there existed different glycolysis-related prognosis genes between COAD and READ.

**Table 3:** Glycolysis-related prognostic genes in COAD and READ by univariate analysis

Cancer type	Gene	HR	95% CIs	*P* value
COAD	*STC1*	1.396	1.053–1.849	0.020
	*ANKZF1*	1.963	1.110–3.470	0.020
	*STC2*	1.251	1.010–1.548	0.040
	*SDHB*	0.490	0.301–0.799	0.004
	*SUCLG2P2*	0.109	0.034–0.345	0.000
	*P4HA1*	1.482	1.062–2.067	0.021
	*PPFIA4*	5.078	2.154–11.970	0.000
	*GPC1*	1.406	1.075–1.839	0.013
	*PCK1*	0.802	0.655–0.981	0.032
READ	*TSTA3*	2.661	1.301–5.443	0.007
	*IDH3A*	0.364	0.167–0.794	0.011
	*PKP2*	0.419	0.238–0.737	0.002
	*ACO2*	0.493	0.290–0.837	0.008

**Table 4:** Glycolysis-related gene risk model for predicting READ prognosis

Cancer type	Gene	*β* (coef)	HR	95% CI	*P* value
COAD	*ANKZF1*	0.543	1.722	0.972–3.049	0.062
	*STC2*	0.257	1.293	1.030–1.624	0.027
	*SUCLG2P2*	− 2.124	0.120	0.035–0.407	0.001
	*P4HA1*	0.359	1.432	1.013–2.025	0.042
	*GPC1*	0.326	1.385	1.040–1.843	0.026
	*PCK1*	− 0.256	0.774	0.632–0.950	0.014
	${\text{Risk}}\;{\text{model}}\;{\text{formula}} = \mathop \sum \nolimits_{i = 1}^{n} {\text{expression }}\;{\text{of }}Gi \times \beta i$ = expression of *ANKZF1* × 0.543 + expression of *STC2* × (0.257) + expression of *SUCLG2P2* × (− 2.124) + expression of *P4HA1* × (0.359) + expression of *GPC1* × (0.326) + expression of *PCK1* × (− 0.256)				

Cancer type	Gene	*β* (coef)	HR	95% CI	*P* value
READ	*TSTA3*	0.752	2.121	1.067–4.218	0.032
	*PKP2*	− 0.725	0.484	0.274–0.856	0.013
	$\mathop {{\text{Risk}}\;{\text{model}}\;{\text{formula}} = \sum }\nolimits_{i = 1}^{n} {\text{expression}}\;{\text{of}}\;Gi \times \beta i$ = expression of *TSTA3* × 0.752 + expression of *PKP2* × (− 0.725)

### The glycolysis-related COAD prognostic model is not appropriate for READ

Above all, we established a risk score model based on the independent prognostic genes of COAD to, respectively, evaluate COAD and READ patient survival. The risk score model formula is as follows: risk score = expression of *ANKZF1* × (0.543) + expression of *STC2* × (0.257) + expression of *SUCLG2P2* × (− 2.124) + expression of *P4HA1* × (0.359) + expression of *GPC1* × (0.326) + expression of *PCK1* × (− 0.256) (Table 4).

**Fig. 3:**
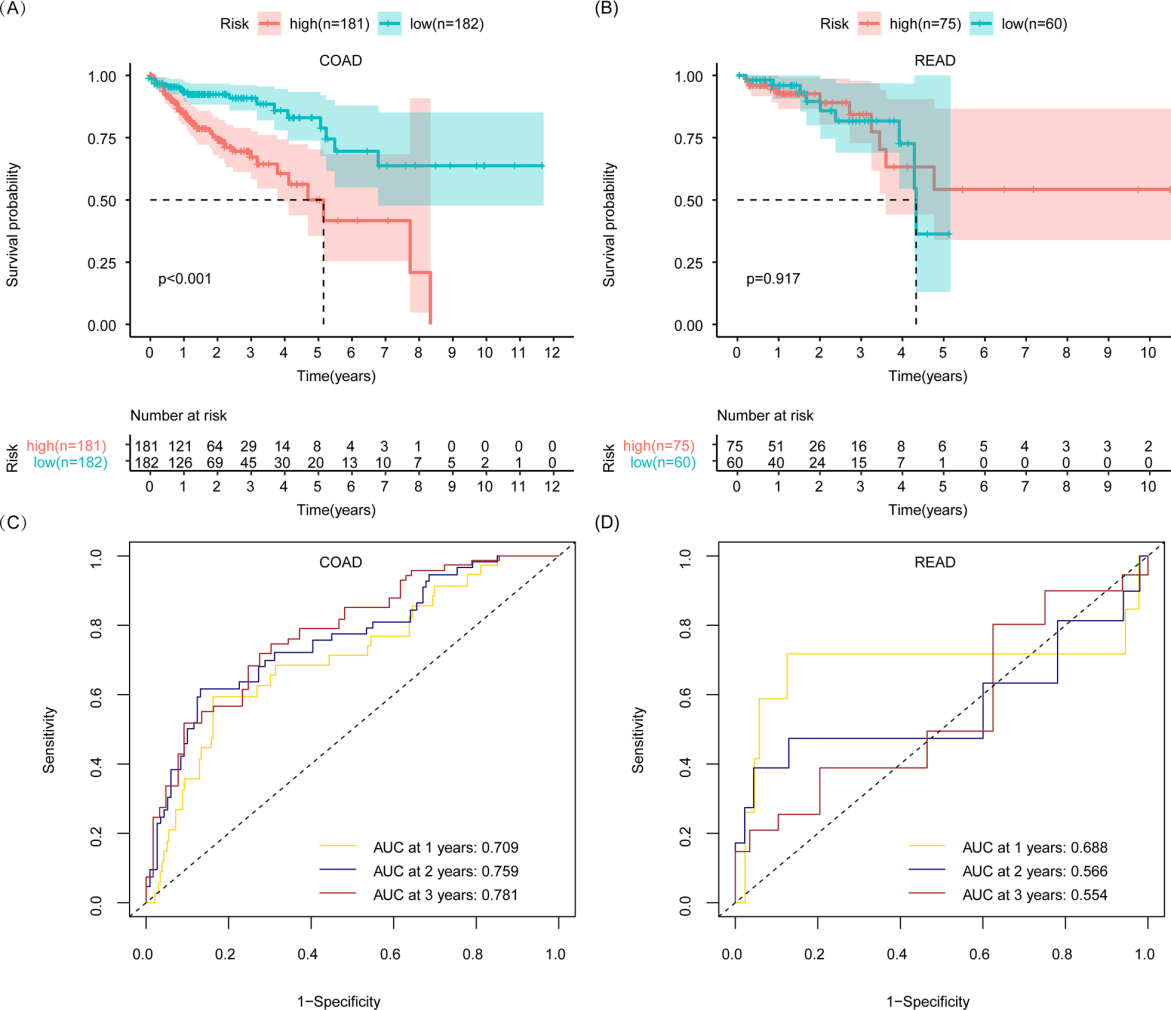
The assessment of glycolysis-related COAD prognostic model in READ and COAD patients. **A** Kaplan–Meier survival analysis on COAD patients between the high-risk and low-risk groups. **B** Kaplan–Meier survival analysis on READ patients between the high-risk and low-risk groups. **C** AUC of the glycolysis-related COAD prognostic model in predicting survival in COAD patients. **D** AUC of glycolysis-related COAD prognostic model in predicting survival in READ patients

COAD patients were divided into a high-risk group and a low-risk group according to the median risk score of the whole COAD patients. We found that COAD patients with a high-risk score had a shorter OS than those with a low-risk score (Fig. 3A). A ROC curve was further drawn to evaluate the reliability of this prognostic model. The AUC of this predictive model accumulated steadily over time and came to 0.781 at 3 years, indicating a satisfactory sensitivity and specificity of this model in predicting survival for COAD patients (Fig. 3C). We wondered whether a risk score model of COAD could distinguish the prognosis of READ patients. The risk score of READ patients was calculated according to the risk model formula of COAD. READ patients were also divided into a high-risk group and a low-risk group according to the median risk score of the whole COAD patients. We found that the glycolysis-related prognostic model of COAD could not distinguish the prognosis of READ patients according to Kaplan–Meier survival analysis (Fig. 3C). Moreover, the AUC of the ROC curve was 0.554 at 3 years in READ (Fig. 3D), which was lower than the AUC in COAD. In addition, READ patients were divided into a high-risk group and a low-risk group according to the median risk score of the whole READ patients based on COAD prognostic model. The survival curves of the high-risk group and low-risk group crossed with the *p* value > 0.05 in READ (Additional file 1: Fig. S2A). These results indicated that the glycolysis-related prognostic model of COAD is not appropriate for READ.

### The glycolysis-related READ prognostic model is suitable for READ

**Fig. 4:**
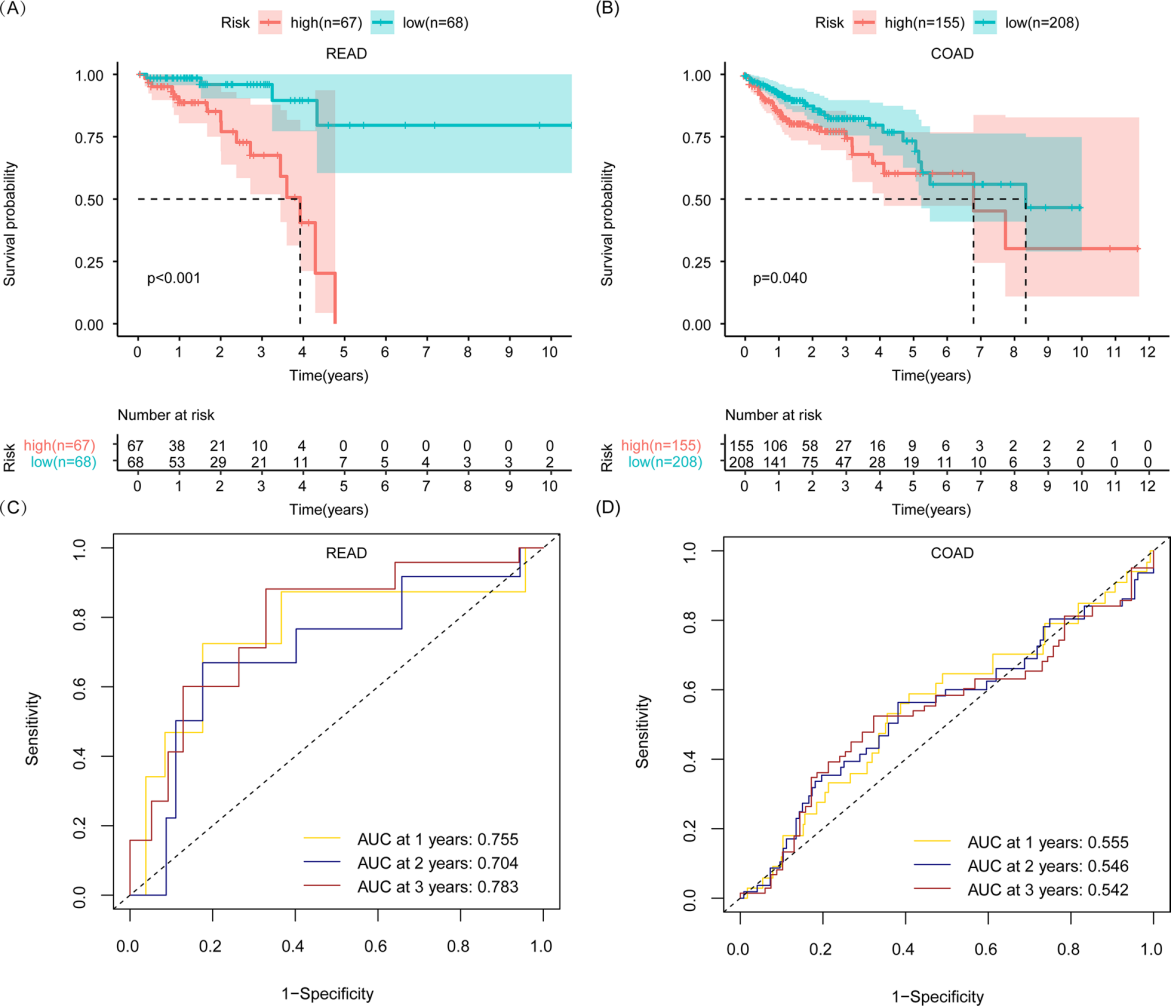
The assessment of glycolysis-related READ prognostic model in READ and COAD patients. **A** Kaplan–Meier survival analysis on READ patients between the high-risk and low-risk groups. **B** Kaplan–Meier survival analysis on COAD patients between the high-risk and low-risk groups. **C** AUC of the glycolysis-related READ prognostic model in predicting survival in READ patients. **D** AUC of glycolysis-related READ prognostic model in predicting survival in COAD patients

Subsequently, we established risk score model based on the independent prognostic gene of READ to, respectively, evaluate READ and COAD patients' survival. The risk score model formula is as follows: risk score = expression of *TSTA3* × (0.752) + expression of *PKP2* × (− 0.725) (Table 4). READ patients were divided into a high-risk group and a low-risk group according to the median risk score of the whole READ patients. We found that READ patients with a high-risk score had a shorter OS than those with a low-risk score (Fig. 4A). A ROC curve was drawn to evaluate the reliability of this prognostic model. The AUC of this predictive model accumulated steadily over time and came to 0.783 at 3 years, indicating a satisfactory sensitivity and specificity for this model in predicting the survival of READ patients (Fig. 4C). We wondered whether the risk score model of READ could distinguish the prognosis of COAD patients. Risk score of COAD patients was calculated according to the risk model formula of READ. COAD patients were also divided into a high-risk group and a low-risk group according to the median risk score of the whole READ patients. We found that the survival curves of the high-risk group and low-risk group crossed despite the *p* value < 0.05 in COAD (Fig. 4B). In addition, the AUC was 0.542 at 3 years in COAD (Fig. 4D), which was lower than the AUC in READ. In addition, COAD patients were divided into a high-risk group and a low-risk group according to the median risk score of the whole COAD patients based on READ prognostic model. The survival curves of the high-risk group and low-risk group still crossed with the *p* value > 0.05 in COAD (Additional file 1: Fig. S2B). These results indicated that the READ risk score model was more appropriate for READ than for COAD.

**Fig. 5:**
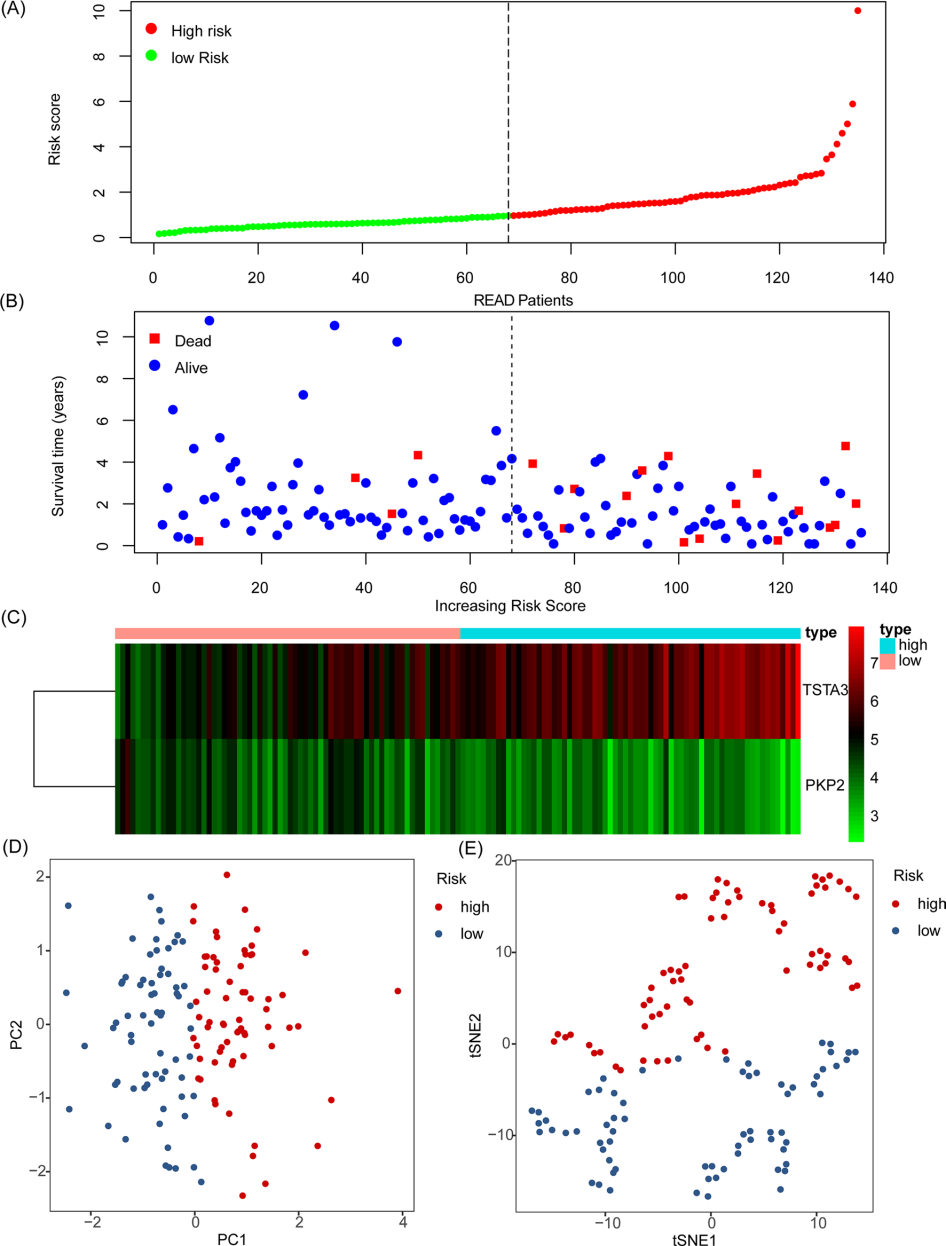
The characteristic of glycolysis-related READ prognostic model. **A** Distribution of risk scores of each READ patient. **B** Correlation between survival time and survival status of each patient. **C** The expression pattern of *TSTA3* and *PKP2* between high-risk and low-risk groups. **D** PCA analysis of READ patient based on glycolysis-related READ prognostic model. **E** t-SNE analysis of READ patient based on glycolysis-related READ prognostic model. The dashed line represents the median risk score in READ patients

To provide visualization of risk score, survival status, and gene expression in READ. The risk score, survival status, and gene expression of the two genes in each READ patient are shown in Fig. 5A–C. PCA and t-SNE analysis confirmed that READ patients in the two subgroups were distributed in discrete directions (Fig. 5D–E).

### Assessment of the glycolysis-related READ prognostic model in READ patients

**Fig. 6:**
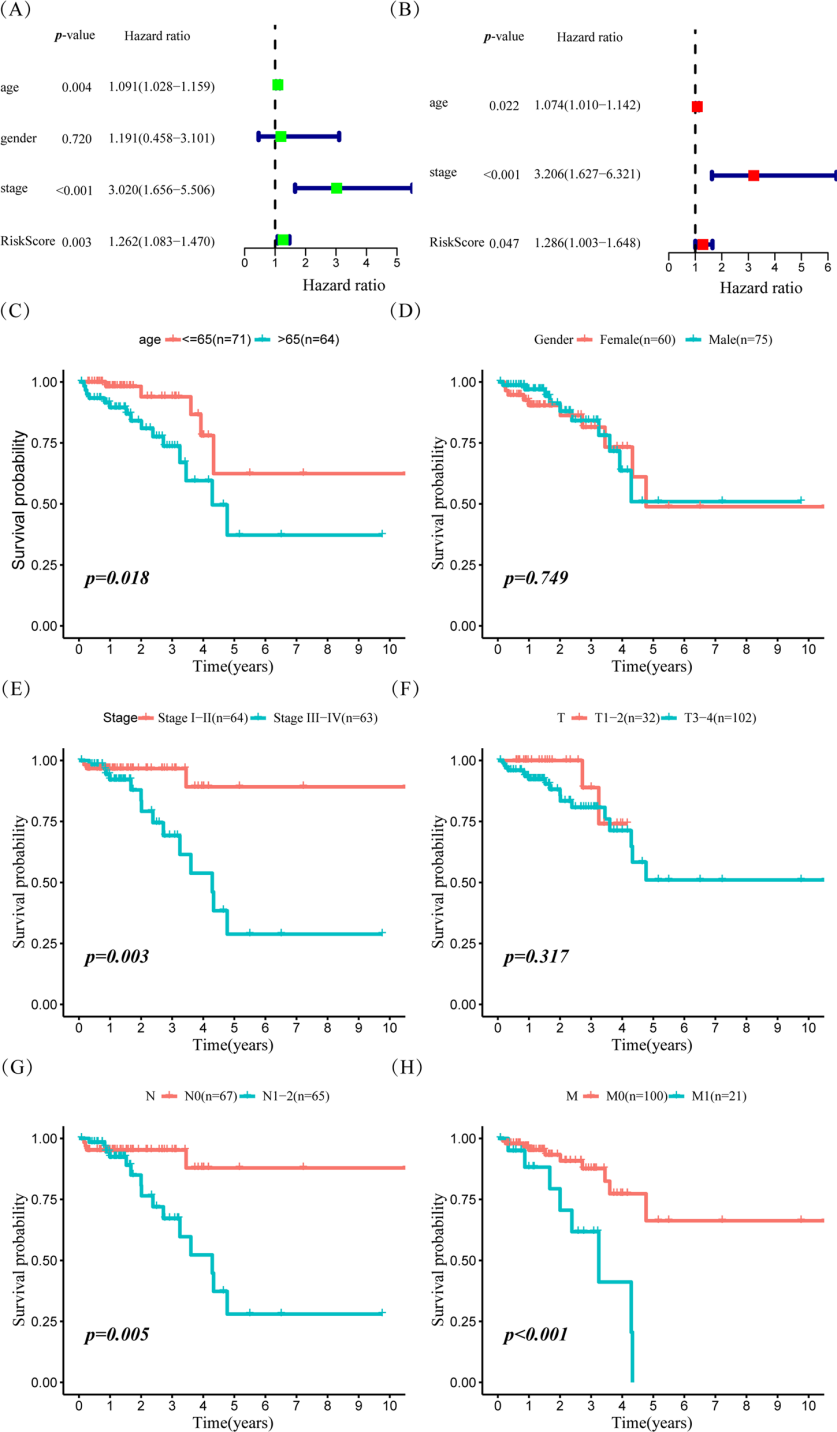
The cox analysis on prognostic model and survival analysis on different clinical features in READ. **A** Forest plot for the risk score model compared with other clinical features in READ patients by univariate analyses. **B** Forest plot for the risk score model compared with other clinical features in READ patients by multivariate analyses. **C**–**H** Kaplan–Meier survival analysis for different clinical features, including age (**C**), gender (**D**), TNM stage (**E**), T stage (**F**), N stage (**G**), and M stage (**H**), in READ patients

To evaluate this prediction model's clinical utility, univariate and multivariate Cox proportional hazards regression analyses were conducted to compare this two-gene risk model with common clinicopathological features. Univariate analysis showed that age, TNM stage, and risk score were associated with rectal cancer patient survival (Fig. 6A). These three factors were further proven to be independent prognostic indicators in the subsequent multivariate Cox analysis (Fig. 6B), indicating that the two-gene risk model can serve as a promising tool for predicting the prognosis of patients with rectal cancer. The Kaplan–Meier curves illustrated that patients with older age, higher N stage, metastasis status, higher TNM stage, and a higher risk score had a poor prognosis. In contrast, gender and T stage had no effect on prognosis of rectal cancer patients (Fig. 6C–H).

**Fig. 7:**
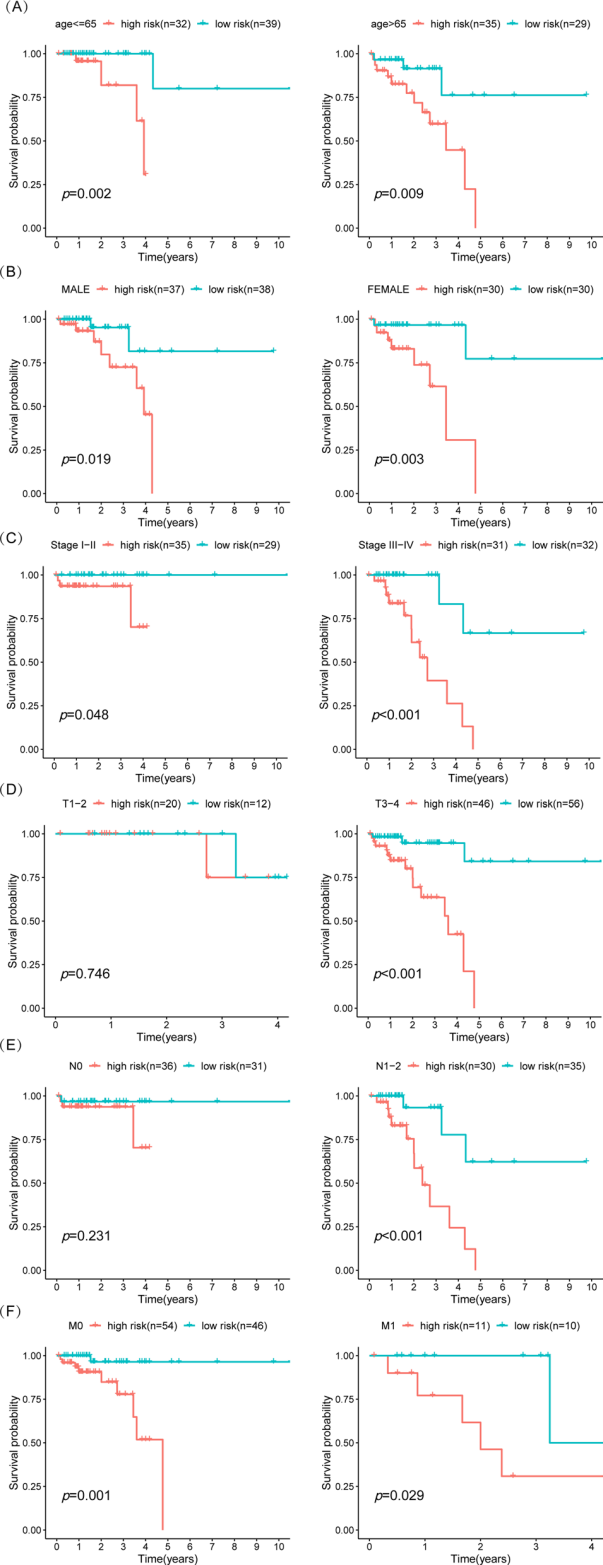
Hierarchical survival analysis of glycolysis-related READ prognostic model in READ. Hierarchical survival analysis on clinical features [age (**A**), gender (**B**), TNM stage (**C**), T stage (**D**), N stage (**E**), and M stage (**F**).]

To further verify the effectiveness of this two-gene model in predicting prognosis in rectal cancers, the patients were classified into different subgroups according to age (≤ 65 years vs > 65 years), gender (female vs male), T stage (T1–2 vs T3–4), N stage (N0 vs N1–2), M stage (M0 vs M1) and AJCC TNM stage (stage I–II vs stage III–IV). They were subsequently divided into the high-risk score and low-risk score groups based on the median risk score. Interestingly, the two-gene risk model still showed robust effectiveness in the different age, gender, M stage, and AJCC stage subgroups (Fig. 7A–F). However, high-risk scores did not suggest a poor prognosis in the N0 stage and T1–2 stage subgroups (Fig. 7A–F), indicating that the risk score model may be a more effective prognostic marker in the late stages of rectal cancer.

### Construction and evaluation of a nomogram incorporating the two-gene risk score model and clinical features

**Fig. 8:**
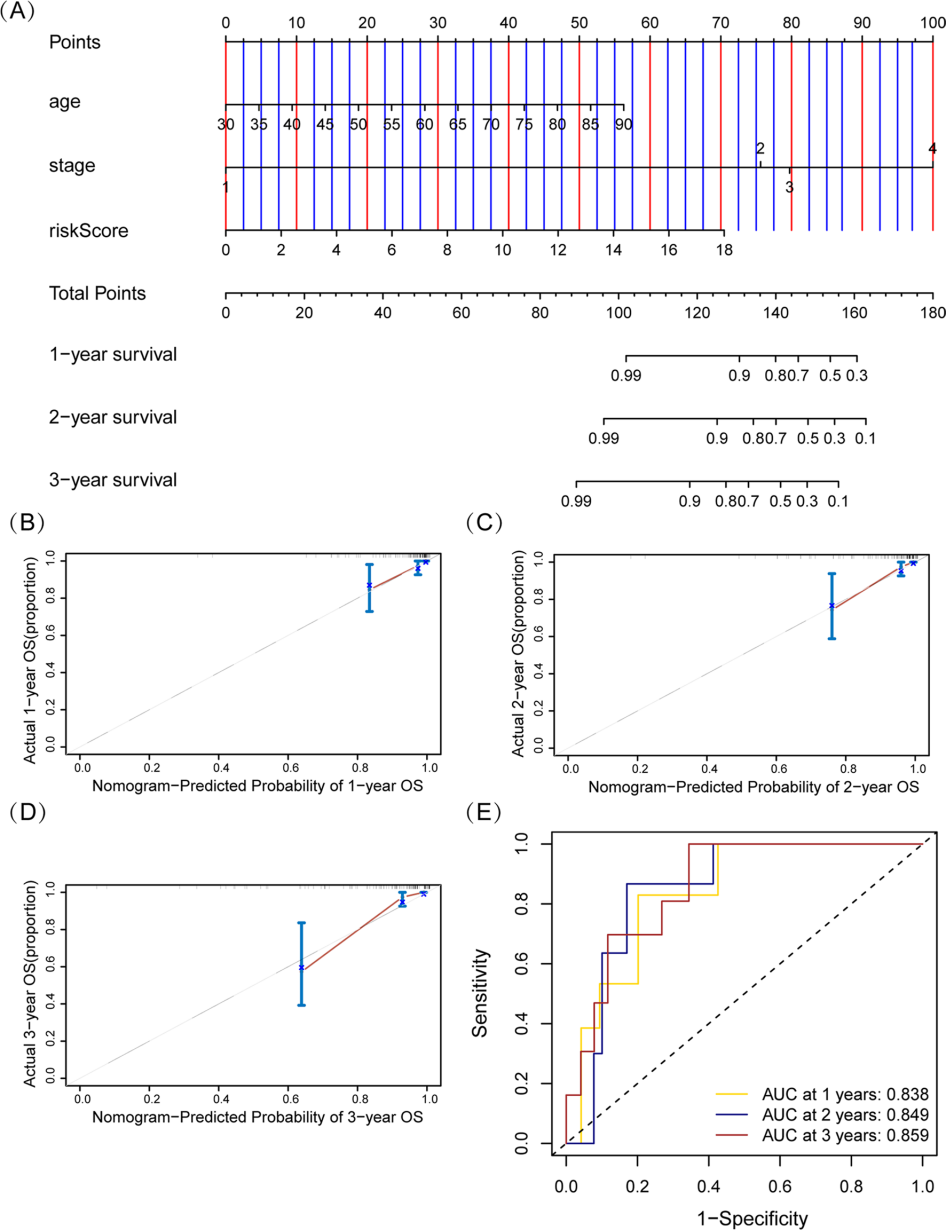
Establishment and evaluation of a nomogram incorporating prognostic model and clinical features in READ patients. **A** Construction of a nomogram combining risk score with clinicopathological characteristics (age and TNM stage). **B**–**D** Calibration plots for the nomogram on 1-, 2‑, and 3‑year survival probability in patients with rectal cancer. **E** Time‑dependent ROC curves for the nomogram on 1-, 2‑, and 3‑year survival probability in patients with rectal cancer

To provide a clinically available and practical tool for oncologists to estimate rectal cancer patients' survival time, we established a nomogram combining risk score with clinicopathological characteristics (age and TNM stage) (Fig. 8A). Calibration plots suggested that the nomogram fitted well compared with the ideal model representing by the 45° line, as shown in Fig. 8B–D. The C-index of the nomogram was 0.852, which indicated that this nomogram had an outstanding stability. ROC curves showed that the AUCs of the nomogram at 1, 2, and 3 years was 0.838, 0.849, and 0.859, respectively, which were better than those of the clinical factors or risk score model alone (Fig. 8E).

### Functional analyses between high-risk and low-risk groups in patients with rectal cancer

**Fig. 9:**
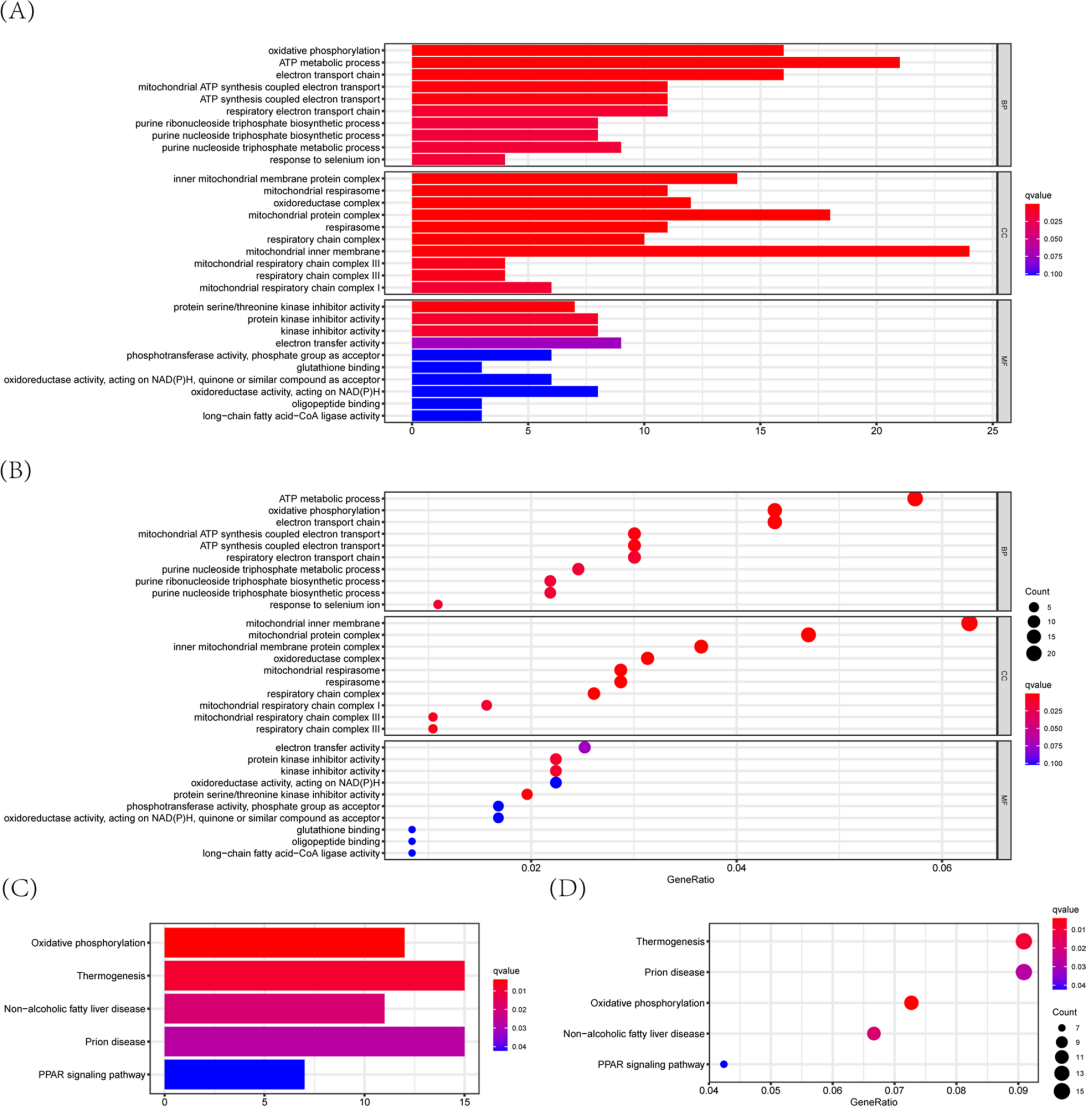
Functional analyses between high-risk and low-risk groups in READ patients. **A**, **B** GO enrichment analysis on DEGs between high-risk and low-risk groups. **C**, **D** KEGG pathway analysis on DEGs between high-risk and low-risk groups

To elucidate the different biological functions or pathways that might play potential roles in the high-risk group and low-risk group, GO enrichment and KEGG analyses were performed using the DEGs (|log2FC| ≥ 0.6, FDR < 0.05) between the two groups. As expected, the DEGs were enriched in several glycolysis-related molecular functions or pathways, such as oxidative phosphorylation (*p* value < 0.05) (Fig. 9A–D). These results indicated that glycolysis-related molecular functions or pathways were indeed different between high-risk group and low-risk group according to glycolysis-related READ prognostic model.

## Discussion

Cancer metabolism, especially glucose metabolism, has drawn great attraction in oncology research during the in recent decades [16–18]. Enhanced aerobic glycolysis, which serves as a metabolic driver, has been proven to promote tumor development and aggressiveness in CRC cells, and further results in resistance to treatment [20, 21]. In our research, we compared glycolysis-related gene sets between COAD and READ. Three gene sets, HALLMARK_GLYCOLYSIS, REACTOME_GLYCOLYSIS, and REACTOME_REGULATION_OF_GLYCOLYSIS_BY_FRUCTOSE_2_6_BISPHOSPHATE_METABOLISM, were significantly enriched in both COAD and READ. We also compared glycolysis-related independent prognostic genes between COAD and READ. We found that six genes including *ANKZF1, STC2, SUCLG2P2, P4HA1, GPC1* and *PCK1* were independent prognostic genes in COAD, while *TSTA3* and *PKP2*, were independent prognostic genes in READ. These results indicated that COAD might have different glycolysis-related prognostic gene signatures than READ. We, respectively, constructed glycolysis-related prognostic models in COAD and READ and, respectively, assessed the efficiency of each model in COAD and READ. We found that the glycolysis-related prognostic model of COAD was not appropriate for READ, while glycolysis-related prognostic model of READ was more appropriate for READ than COAD. These results indicated that COAD might have many different patterns such as genomic signatures, drug efficacy, and prognosis compared with READ.

We also developed a two-gene signature (*TSTA3* and *PKP2*) to predict the prognosis of rectal cancer, which was shown to be an independent prognostic indicator through multivariate Cox analysis. Subgroup analysis confirmed that the READ glycolysis-related prognostic model still showed robust effectiveness in the different age, gender, M stage, and AJCC stage subgroups. Moreover, a nomogram integrating the risk model and clinicopathological factors was depicted to provide clinicians with a practical tool for predicting the prognosis of rectal cancer patients.

Recent research has revealed that traditional clinicopathological factors are inadequate for accurate cancer prognosis predictions [13, 14]. With the rapid development of high-throughput sequencing and the accumulation of cancer genomic data, molecular signatures based on data mining in the public databases have become a reality to predict the outcome of cancer patients, demonstrating higher sensitivity and specificity than the traditional single genes or markers models [11, 12, 15]. Glycolysis-related risk scores have shown excellent performance in predicting prognosis in various solid tumors [14, 22–27]. Liu et al. [23] developed a four‑gene signature (*AGRN*, *AKR1A1*, *DDIT4*, and *HMMR*) related to glycolysis to predict the lung adenocarcinoma patient outcomes that showed desirable accuracy. Another four-gene glycolytic signature (*NUP205*, *NUPL2*, *PFKFB1*, and *PKM*) showed excellent performance in predicting the OS of bladder cancer patients [27]. Similarly, Chen et al. established a risk score model containing seven glycolysis-related genes (*PPARGC1A*, *DLAT*, *6PC2*, *P4HA1*, *STC2*, *ANKZF1*, and *GPC1*) in their recent study that can effectively predict the outcome of colon adenocarcinoma [14]. However, the genes identified in the COAD background in Chen's research are different from the genes identified in our present study, resulting in two different risk models, which further validate the mainstream view that colon cancer and rectal cancer are two diseases [3–5].

Plakophilin-2 (*PKP2*) was initially identified as a desmosomal protein, but further studies have revealed that it is localized in the cytoplasm and nucleus as well [28]. *PKP2* expression is upregulated in many cancers, such as lung [29], ovarian [30], glioma [31], and bladder cancers [32]. Increased *PKP2* expression was also related to a malignant phenotype and poor prognosis in some cancers [29–32]. Arimoto et al. found that *PKP2* enhanced the dimerization of *EGFR* and activated downstream signaling pathways, which further promoted cell proliferation and tumor metastasis [33]. Moreover, *PKP2* may function as a feedback inhibitor of Wnt/β-catenin signaling in CRC stromal fibroblasts and regulate Wnt activity in CRC cells [34]. Our present study found that *PKP2* was downregulated in rectal cancer tissues compared with the adjacent normal tissues. Low *PKP2* expression was correlated with a poor prognosis, indicating a distinct role of *PKP2* in rectal cancer development, which needs to be further investigated.

Tissue-specific transplantation antigen P35B (*TSTA3*), also called GDP-L-fucose synthase, is one of the two rate-limiting enzymes in the de novo synthesis pathway of GDP-L-fucose [35–37]. Serving as a one common glycosylation modification by conjugating fucose to protein‑ or lipid‑bound oligosaccharides, fucosylation is dysregulated in various cancers and is related to carcinogenesis, invasion, and metastasis [38, 39]. The process of fucosylation may provide novel targets for cancer therapeutics [40]. Recently, Zhang et al. [41] found that increased *TSTA3* expression could promote esophageal cancer progression by fucosylation of *LAMP2* and *ERBB2* and could predict a poor prognosis [42]. Wang et al. [43] found that knocking out *TSTA3* in mice could lead to fucosylation deficiency and further result in colitis and adenocarcinoma. In contrast, we discovered that *TSTA3* was upregulated in rectal cancer tissues compared to adjacent normal tissues. High expression was correlated with a poor prognosis, indicating that *TSTA3* may function as a tumor suppressor in rectal cancer. Since *TSTA3* plays different roles in diverse kinds of cancer [42–45], the accurate biological function of *TSTA3* in rectal cancer remains to be elucidated.

The two-gene risk model associated with glycolysis demonstrated effective performance in predicting the clinical outcomes of patients with rectal cancer. However, we could not find a large sample database of high-throughput gene expression READ for validation and we found two more than 100 samples colorectal cancer (CRC) database with microarray gene expression database and OS information in Gene Expression Omnibus (GEO) database for validation. The high-risk group had a higher OS rate than that of low-risk group with the *p* value = 0.029 according to GSE39582 database (Additional file 1: Fig. S3A). The survival curves of the high-risk group and low-risk group crossed with the *p* value > 0.05 according to GSE17538 database (Additional file 1: Fig. S3B). Although the above results were inconsistent with the results of READ, we consider that the glycolysis-related prognostic model of READ might be appropriate for the high-throughput gene expression database not for microarray gene expression database in READ. In future, with new READ high-throughput gene expression database available for public, the glycolysis-related prognostic model of READ might be validated. Besides, the exact biological functions of the predictive genes, *PKP2* and *TSAT3*, remain unclear in rectal cancer and need to be elucidated in our subsequent studies.

## Conclusion

Our study compared the glycolysis-related gene signature between COAD and READ for the first time, found that there existed different glycolysis-related prognostic genes between COAD and READ, and showed that the glycolysis-related prognostic model of COAD was not appropriate for READ. Our study identified two novel glycolysis-related genes (*PKP2* and *TSTA3*) associated with the prognosis of rectal cancer patients, and further established a risk model based on two novel glycolysis-related genes to effectively predict the prognosis of rectal cancer patients. Our study provides insight into the potential role of glycolysis in the development of rectal cancer and requires further investigation.

## Supplementary Information

**Additional file 1**. Supplementary information.

## Data Availability

The data and materials are available under the permission of author.
