# Supplementary material for: A glycolysis-related two-gene risk model that can effectively predict the prognosis of patients with rectal cancer
Source: Hum Genomics. 2022 Feb 2;16:5. doi: 10.1186/s40246-022-00377-0 (PMC8812245; doi:10.1186/s40246-022-00377-0)
Supplement: Supplementary file 1 — Additional file 1. Supplementary information. [file 40246_2022_377_MOESM1_ESM.docx]

Additional file 1: Fig. S1 The workflow for this study.


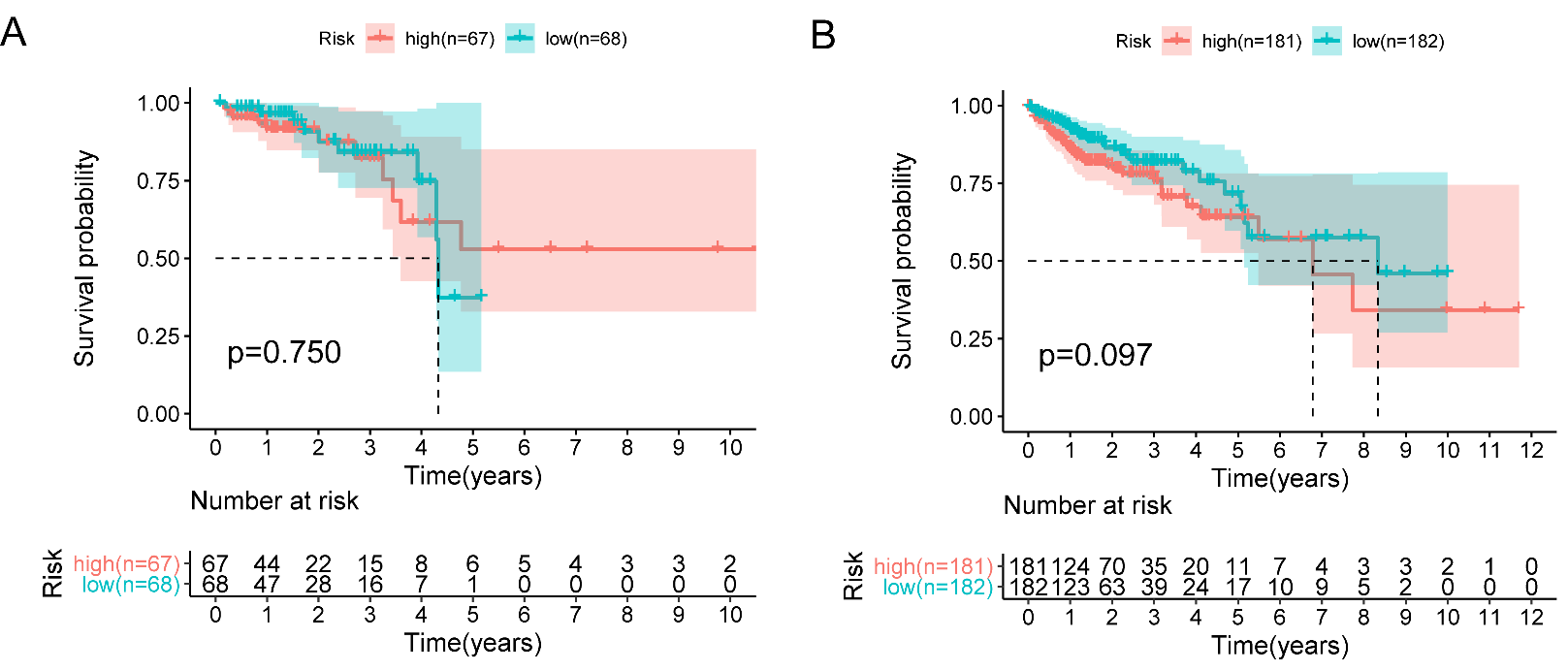


Additional file 1: Fig. S2 The assessment of glycolysis-related prognostic model in READ and COAD patients.(A) Kaplan–Meier survival analysis on READ patients between the high-risk and low-risk groups.Based on glycolysis-related COAD prognostic model, READ patients were divided into a high-risk group and a low-risk group according to the median risk score of the whole READ patients. (B) Kaplan–Meier survival analysis on COAD patients between the high-risk and low-risk groups.Based on glycolysis-related READ prognostic model, COAD patients were divided into a high-risk group and a low-risk group according to the median risk score of the whole COAD patients.


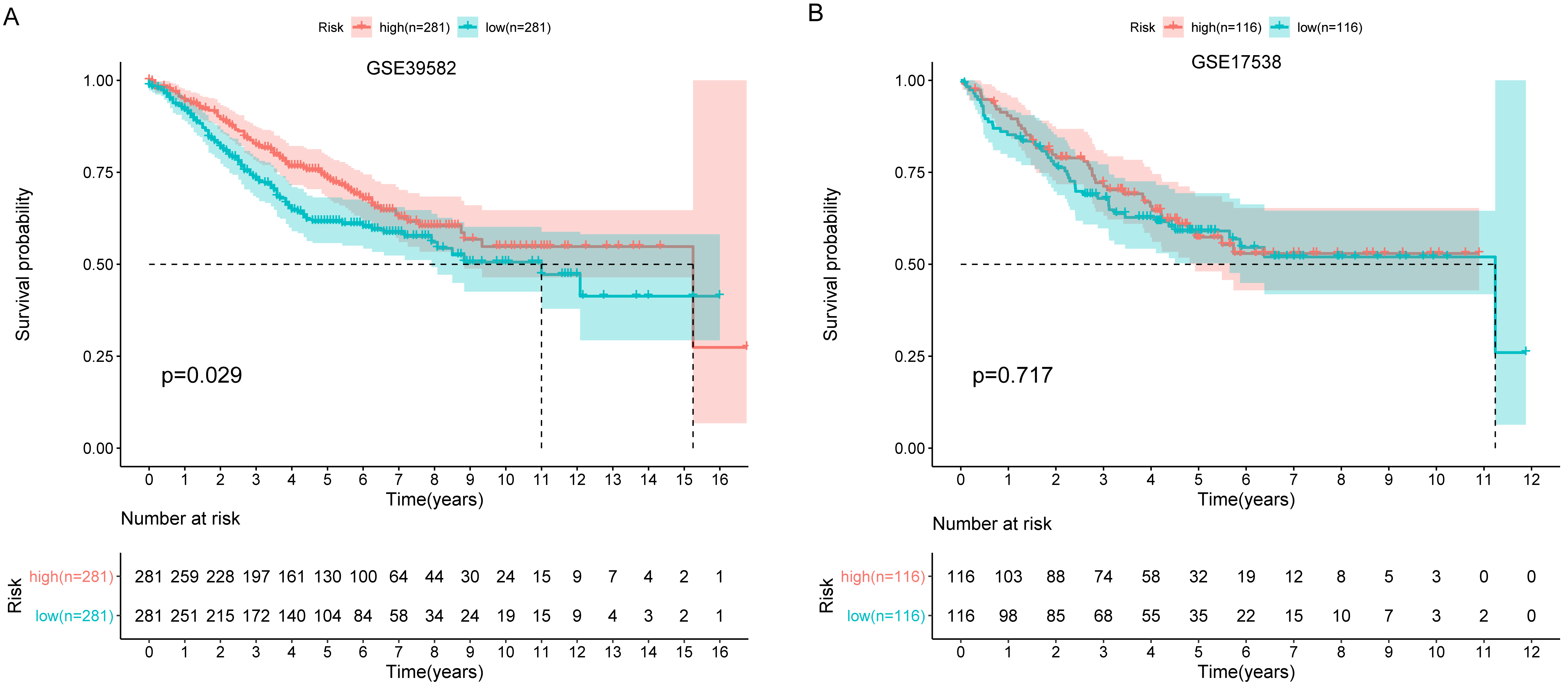
Additional file 1: Fig. S3 The assessment of glycolysis-related READ prognostic model in GEO database.(A) Kaplan–Meier survival analysis on CRC patients between the high-risk and low-risk groups based on GSE39582 database.(B) Kaplan–Meier survival analysis on CRC patients between the high-risk and low-risk groups based on GSE17538 database. Risk score of CRC patients was respectively calculated according to the risk model formula of READ in the GSE39582 and GSE17538 database.CRC patients were respectively divided into a high-risk group and a low-risk group according to the median risk score of the whole CRC patients in the GSE39582 and GSE17538 database.
